# Echo-Planar Imaging for a 9.4 Tesla Vertical-Bore Superconducting Magnet Using an Unshielded Gradient Coil

**DOI:** 10.2463/mrms.tn.2015-0123

**Published:** 2016-03-21

**Authors:** Nao KODAMA, Katsumi KOSE

**Affiliations:** Institute of Applied Physics, University of Tsukuba, 1-1-1 Tennodai, Tsukuba, Ibaraki 305-8573, Japan

**Keywords:** echo-planar imaging, EPI, superconducting magnet, eddy current, gradient coil

## Abstract

Echo-planar imaging (EPI) sequences were developed for a 9.4 Tesla vertical standard bore (∼54 mm) superconducting magnet using an unshielded gradient coil optimized for live mice imaging and a data correction technique with reference scans. Because EPI requires fast switching of intense magnetic field gradients, eddy currents were induced in the surrounding metallic materials, e.g., the room temperature bore, and this produced serious artifacts on the EPI images. We solved the problem using an unshielded gradient coil set of proper size (outer diameter = 39 mm, inner diameter = 32 mm) with time control of the current rise and reference scans. The obtained EPI images of a phantom and a plant sample were almost artifact-free and demonstrated the promise of our approach.

## Introduction

Horizontal bore superconducting magnets are widely used for human whole-body and small animal magnetic resonance imaging (MRI) systems.^1^ In contrast, vertical-bore superconducting magnets are widely used for nuclear magnetic resonance (NMR) spectroscopy for chemical structure analysis. For magnetic resonance (MR) microscopy of small biological samples or live mice, vertical-bore superconducting magnets have several advantages over horizontal-bore ones. The first is that the installation space (e.g., 5-Gauss line area) is much smaller than that of horizontal bore superconducting magnets because the 5-Gauss line generally extends along the axial direction of the cylindrical magnet rather than the radial direction. The second is that the cost of a vertical-bore magnet with high magnetic field strength (e.g., 9.4–16 Tesla) is much lower than that of a horizontal-bore superconducting magnet with an identical magnetic field strength because high-field vertical-bore magnets are massively produced for NMR spectrometers.

However, the vertical-bore superconducting magnets available in the commercial market have a serious problem; that is, their room temperature bore is made of pure Cu metal, mainly because of the low magnetic field susceptibility (∼ −1.0 × 10^−6^) of pure Cu and its ease of fabrication (Hirose R; private communication). Because the room temperature bore of horizontal-bore superconducting magnets is usually made of nonmagnetic stainless steel or sometimes fiber-reinforced plastic to reduce eddy currents induced by current switching of the gradient coils, the Cu bore of vertical-bore magnets clearly presents a serious disadvantage in MRI applications.

To reduce eddy currents on the magnetic bore, the use of actively shielded gradient coils is the most straight-forward solution.^2^ However, for small-bore magnets such as vertical standard [inner diameter (i.d.) ∼54 mm] bore magnets for NMR spectroscopy actively shielded gradient coils substantially reduce the imaging volume because they generally have more than twice the thickness of unshielded gradient coils. Therefore, the use of unshielded gradient coils can be a promising approach for MRI systems using a small-bore superconducting magnet.

Echo-planar imaging (EPI) is one of the fastest imaging methods and requires both fast switching of intense field gradients and a highly homogeneous magnetic field.^3,4^ In particular in a high magnetic field such as 9.4 Tesla, sample-induced magnetic field inhomogeneity, as well as the intrinsic magnetic field inhomogeneity, becomes a major obstacle for EPI, because the data-acquisition window of several tens of milliseconds requires magnetic field inhomogeneity at the order of 0.1 ppm. In addition, the switching of the intense field gradients using an unshielded gradient coil set induces large eddy currents with various time constants that produce serious artifacts in EPI images. Therefore, the development of EPI sequences in such a high magnetic field using an unshielded gradient coil is challenging and, to our knowledge, no report has been published on this topic. In this study, we developed one shot EPI (64 × 64 image matrix) and multishot EPI sequences (128 × 128, 256 × 256, and 256 × 256 × 16 image matrices) for a 9.4 Tesla vertical standard bore superconducting magnet using an unshielded gradient coil and solved the problems by using a proper size gradient coil optimized for live mice imaging with current rise-time control and reference scans.

## Materials and Methods

### MRI system using a 9.4 Tesla vertical-bore superconducting magnet

**Fig. 1.**
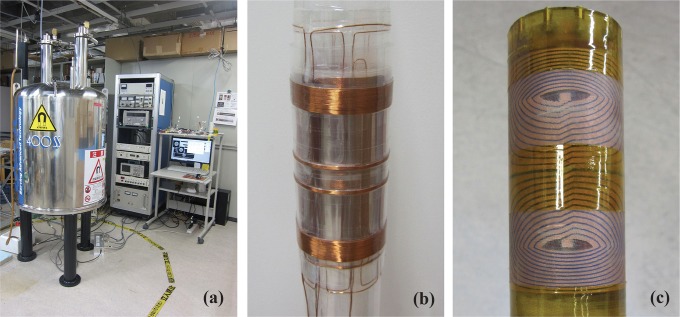
(**a**) Overview of the magnetic resonance imaging system using a 9.4 Tesla vertical standard bore (∼54 mm) superconducting magnet, (**b**) the room temperature shim coil, and (**c**) unshielded gradient coil. The radius of the 5-Gauss line was about 1 m.

Figure 1 shows the MRI system, the room temperature shim coil set, and the unshielded gradient coil set used in this study. The vertical-bore superconducting magnet (JMTC-400/54/SS, JASTEC, Kobe) had the following specifications; room temperature bore 53.84 mm in diameter made of Cu, with a magnetic field strength of 9.4 Tesla and about 1 ppm magnetic field inhomogeneity over a cylinder 17 mm in diameter × 36 mm in length, and with less than 4 Hz/h magnetic field stability.

The room temperature shim coil set was designed after Roméo and Hoult^5^ and had five second-order shim coil channels with spatial symmetry proportional to *xy*, *x*^*2*^–*y*^*2*^, *z*^*2*^, *xz*, and *yz*. These coils were wound with 0.4 mm diameter polyurethane-coated Cu wire over an acrylic pipe (outer diameter (o.d.) = 44 mm, i.d = 40 mm) and driven by constant current power supplies (maximum current: ±1A).

The gradient coil set was originally designed for live mice imaging in the standard bore superconducting magnet. Therefore, the inner bore size was initially determined as 32 mm to accommodate a live mouse, and the outer diameter was determined as a consequence of the gradient coil element design. The gradient coil elements (Gx, Gy, and Gz) were made from Cu rods as follows: gradient coil patterns designed with the target field method^6^ were cut on the surface of the rods using a five-axis numerically controlled lathe; the trenches made by the lathe were filled with epoxy resin including ceramic powder, and were finally bored using a lathe to cylindrical shapes about 0.8 mm thick. Three cylindrical gradient elements were assembled to a rigid gradient coil set (o.d. = 39 mm, i.d. = 32 mm, thickness = 3.5 mm) using a vacuum impregnation technique. The resistivity and current efficiency of the gradient coil elements were 122.2, 101.7, and 100.9 mΩ, and 19.0, 19.2, and 33.7 mT/m/A, for Gx, Gy, and Gz, respectively. We call this gradient coil set “the standard size gradient coil set” to compare this with a larger size gradient coil set to be described in the next subsection. The gradient coil elements were driven by three channel gradient drivers (maximum current: ±20A). A home-built eight-rung birdcage coil, 19.5 mm in diameter and 16.5 mm in length tuned to 400.4 MHz was used for both radio frequency (RF) excitation and signal reception.

A digital MRI transceiver (DTRX6, MRTechnology, Tsukuba, Ibaraki) controlled by a personal computer (PC) controller^7,8^ was used for generation of pulse sequences and NMR signal acquisition timing control, which was actually controlled by a flexible data acquisition software program (Sampler 7D, MRTechnology) running under Windows 7 operating system (Microsoft, Redmond, WA, USA). The PC controller has great capability in gradient wave generation because every pulse sequence event [e.g., RF amplitudes (16-bit in quadrature), gradient amplitudes (16-bit for each channel), and data acquisition control] can be controlled by a 128-bit word issued every 1 μs.^7^

### Measurements of eddy currents generated by gradient coils

Eddy currents generated by current switching of the gradient coils were measured using gradient echoes generated after intense readout gradient pulses applied before the RF pulse.^9,10^ The time between the RF pulse and the falling edge of the readout gradient pulse varied (0.1, 0.2, 0.3, …10.0 ms) and the temporal shift of the gradient echo peak was measured repeatedly. Time constants of the eddy currents were calculated by fitting the decaying curves of the eddy currents. To compare the performance of the standard size gradient coil set, eddy currents of an unshielded gradient coil set with larger diameters (45 mm o.d. and 41 mm i.d.) wound by 0.5 mm diameter Cu wire were measured in the same way.

### One-shot EPI sequence and its image reconstruction method

**Fig. 2.**
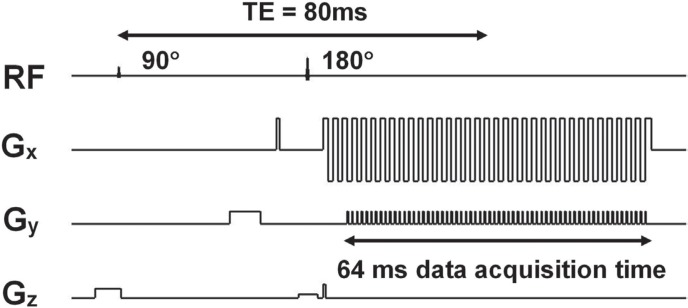
One shot echo-planar imaging sequence for a 64 × 64 image matrix. The rise time of the gradient waveform was controlled to 0.2 ms using a personal computer controller. The radio frequency (RF) pulses were in sinc function with seven side lobes (±4π). Data acquisition started after five times of the initial readout gradient switching to avoid contamination of unsteady signals. TE, spin-echo time.

Fig. 2 shows a one-shot two-dimensional (2D) spin-echo EPI pulse sequence for a 64 × 64 pixel image [slice thickness = 2 mm, field of view (FOV) = 15.36 mm^2^] developed in this study. The spin-echo time (TE), the time between multiple gradient echoes, and total data-acquisition time were 80, 1, and 64 ms, respectively. To reduce eddy currents, the rise time (0% to 100%) of the readout gradient current (maximum strength = ±153 mT/m) was controlled to 0.2 ms by the PC controller. Data acquisition started after five times of the initial readout gradient switching to avoid contamination of unsteady signals. Although the Nyquist frequency for the NMR signal was 100 kHz (dwell time of the signal sampling = 10 μs), we used double oversampling (dwell time = 5 μs) to detect the peak echo time more precisely and reduce resampling error.

As shown in “Reference scan,” subsection of “Results” section, because the relative peak positions in the switching period (1 ms) of the readout gradients and the phases of the multiple gradient echoes observed in the EPI sequences frequently deviate from ideal values, a reference EPI scan that measures the NMR signal without the phase encoding gradient was acquired.^4,11^

The reference scan used in this experiment and known by many researchers from 1980s^11^ is based on the principle that the EPI k-trajectory repeatedly passes through the origin of the k-space when the readout gradient is applied and the phase encoding gradient is switched off. Because all the magnetization vectors refocus at the origin of the k-space, the (gradient) echo peaks are repeatedly observed. Ideally, the echo peak positions are equally spaced in time and the phases of the echoes are identical. However, because eddy currents generated by the intense readout gradient switching usually generate long-term change of the readout gradient and instantaneous B_0_ shift (spatially homogeneous component of the eddy field) after the switching, fluctuation of the peak positions and alternative change of the echo phase are observed. These positional fluctuation and phase shift can be corrected using those values measured beforehand (reference scan). Therefore, the EPI image was reconstructed using 2D fast Fourier transform after the sampling points were resampled along the readout direction to align the peak echo time in the switching period and the phases of the gradient echoes were corrected using phases at the peak positions.

### Multishot and three-dimensional (3D) EPI sequences

**Fig. 3.**
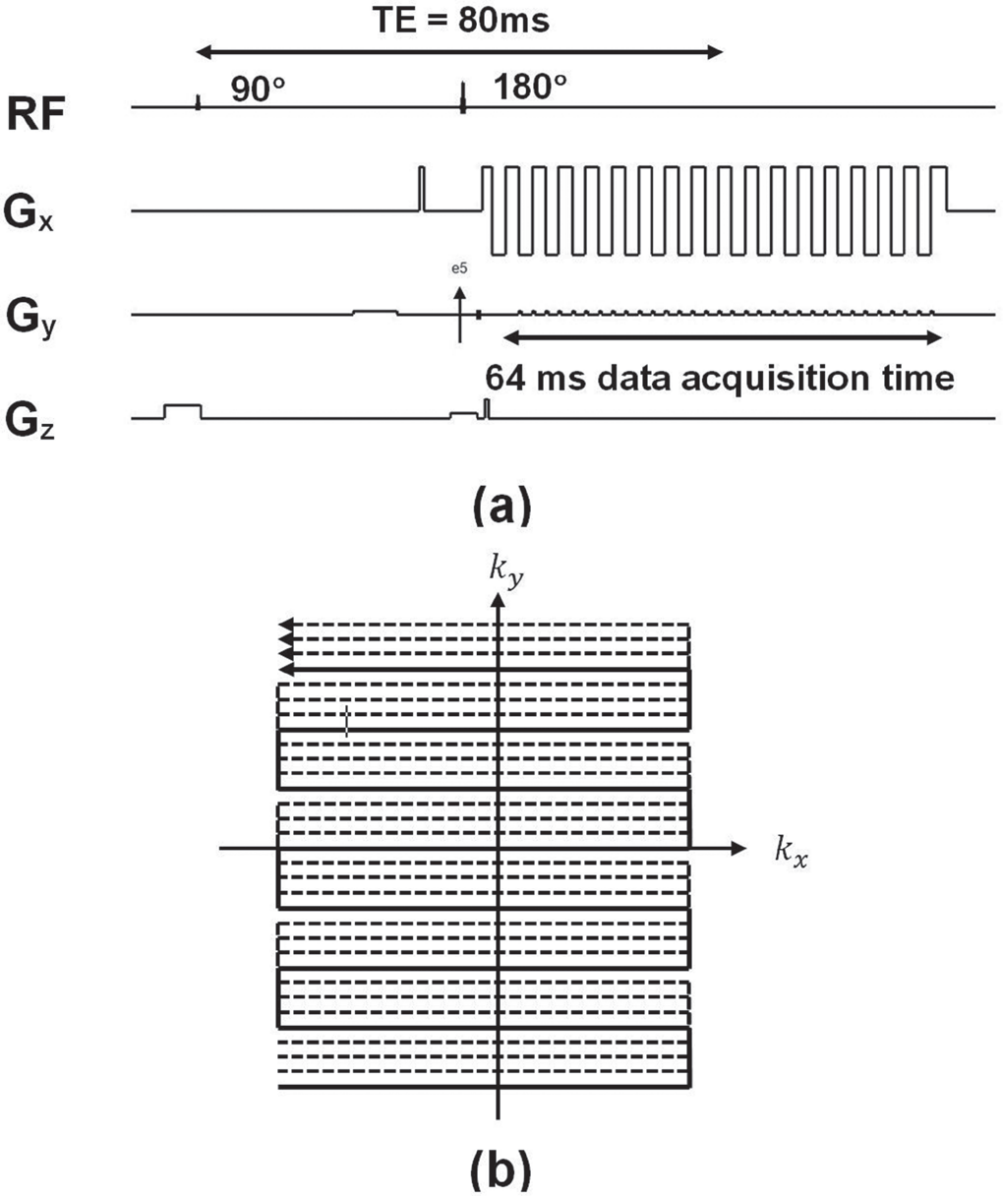
(**a**) Multishot echo-planar imaging (EPI) sequence for a 128 × 128 image matrix. (**b**) Four interleaved trajectories in the k-space for the 128 × 128 multishot EPI sequence. The interleaved trajectory shown in the *solid line* was shifted using the phase encoding gradient applied before the readout gradient. “e5” in the Gy waveform indicates the phase encoding gradient to be used to shift the EPI trajectory. RF, radio frequency; TE, spin-echo time.

For multishot EPI sequences with 128 × 128 and 256 × 256 pixel images, the time between multiple gradient echoes was doubled to 2 ms and quadrupled to 4 ms to keep the total data-acquisition time (64 ms) constant. To slide the interleaved trajectory of the multishot EPI sequence along the phase encoding direction, an additional phase encoding gradient was applied before the signal readout (Fig. 3). For the multishot EPI sequence with a 128 × 128 pixel image, 32 echoes were used in the one-shot sequence and four shots with the additional phase encoding gradient were used. Therefore, the measurement time for one image was 4 TR (repetition time of the sequence). For the multishot EPI sequence with a 256 × 256 pixel image, 16 echoes were used in the one-shot sequence, and 16 shots with the additional phase encoding gradient were used. Therefore, the measurement time for one image was 16 TR.

**Fig. 4.**
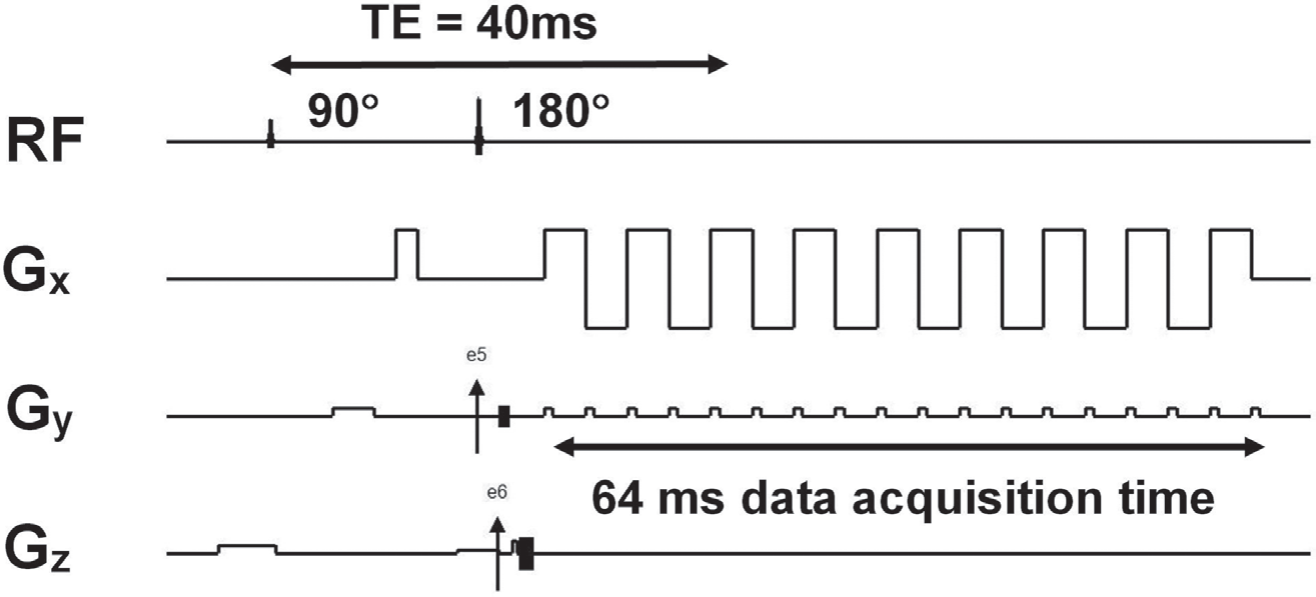
Multishot three-dimensional echo- planar imaging (3D EPI) sequence for a 256 × 256 × 16 image matrix. Two hundred and fifty-six shots are required for the 3D acquisition. “e5” in the Gy waveform indicates the phase encoding gradient to be used to shift the EPI trajectory. “e6” in the Gz waveform indicates the phase encoding gradient along the slicing plane. RF, radio frequency; TE, spin-echo time.

For the multishot 3D EPI sequence, another additional phase encoding gradient (Gz) was applied perpendicular to the 2D imaging plane (Fig. 4). For the multishot 3D EPI sequence with the 256 × 256 × 16 pixel image, 16 echoes in the one-shot sequence, 16 in-plane phase encoding steps, and 16 phase encodings perpendicular to the plane were used. Therefore, 16 × 16 = 256 shots were used, for which measurement time for one image dataset was 256 TR. The FOV was (15.36 mm)^3^.

The 2D and 3D multishot EPI images were reconstructed after signal correction using corresponding reference scans as described in the previous subsection.

### Samples for sequence evaluation

A water phantom was made by storing 19 closely packed glass capillaries (o.d. = 1.4 mm, i.d. = 0.9 mm, length = 12 cm) in an NMR sample tube (o.d. = 10.0 mm, i.d. = 9.0 mm, length = 13 cm) filled with CuSO_4_ doped water solution (T_1_ ∼ 200 ms). A stem of celery (length = 8 cm) was used to demonstrate the possibility of EPI sequences for biological applications.

### Magnetic field shimming

Magnetic field shimming for EPI was performed using a spin-echo signal of a 2D conventional spin-echo sequence (slice thickness = 2 mm) with TE = 80 ms and 64 ms data-acquisition window. Currents of the five-channel second-order shim coils and offset currents of the three-channel field gradients (Gx, Gy, and Gz) were repeatedly and manually changed to maximize the decay constant ( ${\text{T}}_{2}^{*}$ ) of the spin-echo signal.

## Results

### Eddy currents generated by the gradient coils

Tables 1 and 2 show amplitudes and time constants of the major eddy current gradient field components measured for the gradient coils Gx, Gy, and Gz.^10^ For the standard size gradient coil set, Table 1 clearly show that the time constants were categorized into two groups with ∼0.3 ms and ∼2 ms, and the amplitudes were 5–10% of the applied field gradients. For the larger size gradient coil set, the amplitudes were about two times larger and the time constants of the “eddy current gradient field 2” were much longer (∼10 ms) than those of the standard size gradient coil set.

### Reference scan

**Fig. 5.**
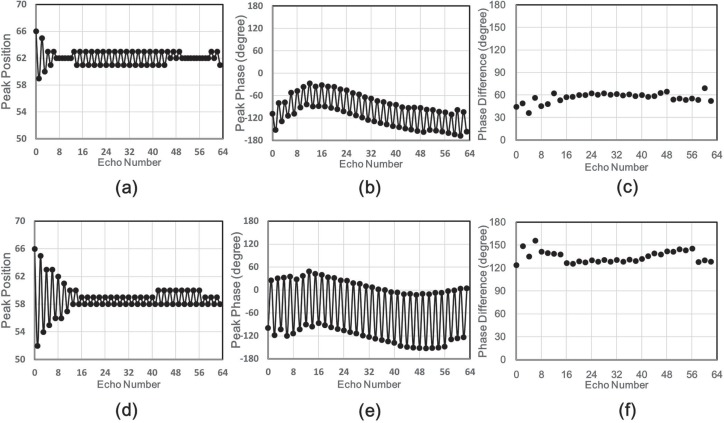
Peak position [(**a**) and (**d**)] represented by the sampling points (5 ms interval) and peak phase [(**b**) and (**e**)] of the multiple gradient echoes generated by Gx [(**a**) and (**b**)] and Gy [(**d**) and (**e**)] readout gradients in one-shot EPI sequences measured for the water phantom. The phase differences in [(**c**) and (**f**)] mean those between two successive multiple gradient echoes for Gx and Gy readout gradients.

Figure 5(a, d) shows the relative positions represented by the sampling points (5 μs interval) in the switching interval (1 ms) of the readout gradients of multiple gradient echo peaks generated by Gx and Gy readout gradients in one-shot EPI sequences measured for the water phantom. The peak positions fluctuated back and forth for several points (∼5 ms) and about 15 points (∼15 ms) for the Gx and Gy readout gradients around the starting part of the successive echoes, but the fluctuation soon (∼5 and ∼15 ms) decreased to nearly zero.

Fig. 5(b, e) shows the phases of the multiple gradient echo peaks generated by Gx and Gy readout gradients in the one-shot EPI sequences measured for the water phantom. The phases of the echo peaks were changed up and down by B_0_ eddy currents produced by the readout gradient switching because the sign of the B_0_ changed alternatively. Fig. 5(c, f) shows differences between phases of two successive multiple gradient echoes. The nearly constant phase differences (∼60° for Gx and ∼140° for Gy) show that the phase changes were determined by the B_0_ component of the eddy current produced by the readout gradient switching.

### EPI images

**Fig. 6.**
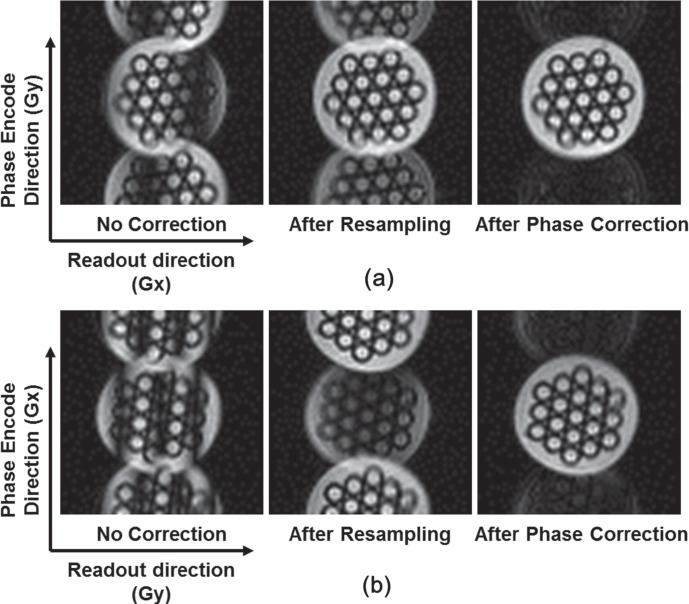
Reconstructed images acquired with the one-shot EPI sequences using (**a**) Gx and (**b**) Gy readout gradients without data correction (left), after resampling using the echo peak timing (middle), and after resampling and phase correction (right) using the echo phase values of the reference scans. N/2 artifacts are barely observed.

Figure 6 shows reconstructed images acquired with the one-shot EPI sequences using Gx and Gy readout gradients without data correction, after resampling along the readout direction, and after both resampling and phase correction. As described above, the resampling and the phase correction were performed using the relative peak positions in the switching interval and the phase values of the multiple gradient echoes of the reference scan data. As shown in the corrected images, sinusoidal intensity variations along the readout directions are corrected by the resampling and the N/2 ghost was corrected using the phase correction. The ratios of the averaged image intensities of the water phantom to those of the N/2 ghost artifacts in Fig. 6(c, f) were reduced to 7.96 and 6.93, which were calculated in square regions including the ghost artifacts and central ghost free regions. No RF inhomogeneity was observed as shown in the corrected images, because the wavelength of 400 MHz electromagnetic wave is about 8 cm in the water phantom (relative permittivity: ε_r_ ∼80), which is much longer than the size of the phantom (∼1 cm).

Because ${\text{T}}_{2}^{*}$ of the spin-echo signal of the water phantom in the selected slice was about 40 ms, the full width at half maximum of the frequency spectrum was about 8 Hz. The image distortion caused by the magnetic field inhomogeneity in the slicing plane observed for the EPI images was less than a few pixels, which demonstrated the magnetic field inhomogeneity was less than about several tens of Hz or 0.1 ppm in 400 MHz, because the frequency resolution along the phase encoding direction was 1/(64 ms) or 15.625 Hz/pixel.

**Fig. 7.**
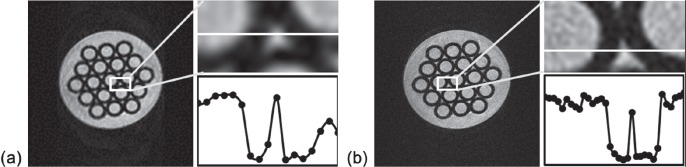
Reconstructed images and image intensity profiles acquired for multishot EPI sequences: (**a**) 128 × 128 and (**b**) 256 × 256 pixel images. The profiles where the image intensities rise from zero to the maximum by about one pixel width demonstrate that the theoretical resolution is achieved for both images.

Fig. 7 shows reconstructed images acquired with the multishot EPI sequences for 128 × 128 and 256 × 256 pixel images. Because TR was 400 ms, the measurement times for these images were 1.6 and 6.4 s. As shown in the profiles, the theoretical spatial resolution 120 μm^2^ and 60 μm^2^ was achieved for both images.

**Fig. 8.**
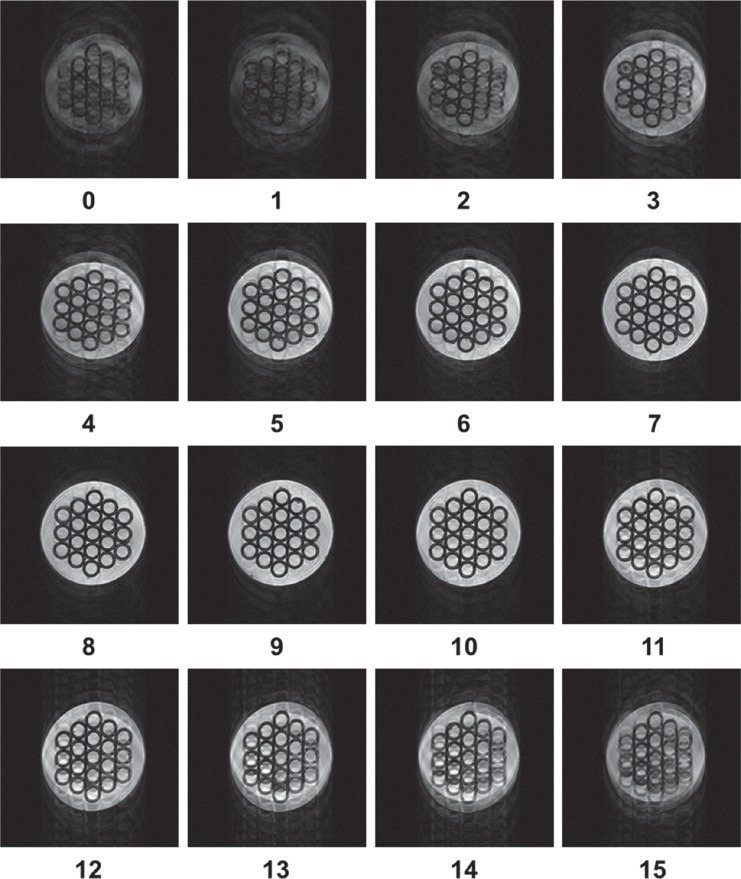
Image dataset of the water phantom acquired with the multishot 3D EPI sequence for a 256 × 256 × 16 pixel image. The reference scan for the 2D 256 × 256 multishot EPI image (slice thickness = 2 mm) was used to perform the resampling and the phase correction. 3D EPI, three-dimensional echo-planar imaging.

**Fig. 9.**
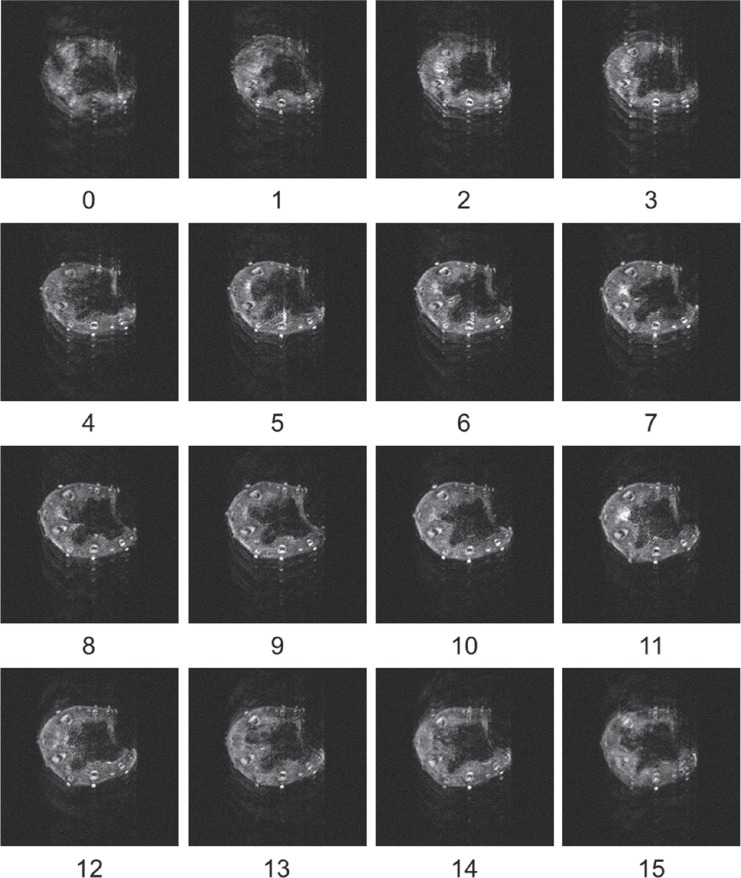
Image dataset of a stem of celery acquired with the multishot 3D EPI sequence for a 256 × 256 × 16 pixel image. The reference scan for the 2D 256 × 256 multishot EPI image (slice thickness = 2 mm) of the water phantom was used to perform the resampling and the phase correction. 3D EPI, three-dimensional echo-planar imaging.

Fig. 8 shows an image dataset of the water phantom acquired with the multishot 3D EPI sequence for a 256 × 256 × 16 pixel image. Because the TR was 400 ms, the measurement time for the image dataset was 102.4 s. Fig. 9 shows an image dataset of a stem of celery acquired with the multishot 3D EPI sequence for a 256 × 256 × 16 pixel image. Because the TR was 800 ms, the measurement time for the image dataset was 204.8 s. The N/2 artifacts were successfully corrected by the reference scan in the central slices (6–10 in Fig. 8, 10–11 in Fig. 9). However, because eddy currents varied spatially, the artifacts were not corrected for the slices near the edge of the images.

## Discussion

### Eddy current effects observed in the reference scan

There are many magnetic field components with different spatial symmetry and different time constants that are generated by eddy currents induced by current switching of gradient coils.^9^ For example, current switching of a gradient coil generates a B_0_ shift (spatially homogeneous component), magnetic fields proportional to the *x*, *y*, and *z* coordinates (first-order components), and those proportional to *xy*, *x*^*2*^–*y*^*2*^, *z*^*2*^, *xz*, and *yz* (second- order components). However, numerous studies have shown that the primary components of the magnetic field induced by current switching of a gradient coil are B_0_ and the field component with the same spatial symmetry as the gradient coil, the so-called eddy current gradient field.^9^ Therefore, we consider the fluctuations of the relative peak positions and phase changes of the multiple gradient echoes observed in the reference scan using the B_0_ shift and the eddy current gradient fields.

The eddy current *e*(*t*) for a step function of a gradient field with one of the spatial components can be expressed as

$$e(t)=\sum_{k=1}^{n}c_{k}\text{exp}(-t/\tau_{k}),$$

where *c_k_* and *τ_k_* are amplitudes and time constants of eddy current components.^9^ The magnetic field *g*(*t*) generated by the eddy currents (eddy current field) induced by switching of the gradient G field is given by

$$g(t)=-\frac{dG}{dt}*e(t),$$

where the symbol * represents convolution. If the gradient field G is applied as a step function, the eddy current field *g*(*t*) can be expressed as

$$g(t)=\sum_{k=1}^{n}g_{k}\text{exp}(-t/\tau_{k}),$$

where *g_k_*’s are amplitudes of the eddy current fields, because *dG*/*dt* behaves like the δ function. In the discussion below of the multiple gradient echoes of the EPI sequences, we assume the switching of the readout gradients (switching time = 0.2 ms) as the step function.

As shown in Table 1, time constants (τ*_k_*) of the major eddy current gradient field components for Gx, Gy, and Gz of the standard size gradient coils were categorized into two groups with ∼0.3 ms and ∼2 ms, and the amplitudes (*g_k_*) were 5%–10% of the applied field gradients. By using these two-component eddy current gradient fields, the decaying fluctuation of the relative peak positions observed around the starting part as (Fig. 5) can be considered in the next two paragraphs.

In the oscillating readout gradients Gx and Gy with 1 ms switching interval, the eddy current gradient fields with ∼0.3 ms time constants deformed the actual waveforms of the gradient fields by about 10% during the switching of the gradients, but the effects decayed to nearly zero in the switching interval, because exp(−1.0/0.3) is about 4%. In contrast, the eddy current gradient fields with ∼2 ms time constants affected the gradient fields not only in the same switching interval but also in the several later switching intervals.

In the oscillating readout gradients, the gradients rose from zero to the maximum at the beginning of the readout gradients and started switching from the maximum to minimum and from the minimum to maximum as shown (Fig. 2). Because the gradient switching was asymmetric about the base line of the gradients (Gx = 0 and Gy = 0), the effects of the eddy current gradient fields caused by the initial positive readout gradients remained for some time (∼5 ms for Gx and ∼15 ms for Gy), corresponding to the decay time of the longer eddy current field gradients. Therefore, a steady-state EPI signal was obtained at ∼10 ms for Gx and ∼20 ms for Gy readout gradients after the start of gradient switching, because five-times initial readout gradient switching was not used for the data acquisition.

As shown in Fig. 5(b, c, e, f), the phase shifts of the echo peaks consisted of two components. The first major one was ∼60° and ∼140° for Gx and Gy readout gradients caused by the B_0_ shift of the eddy currents induced by the readout gradient switching. Although we did not measure the time constants of the B_0_ shift, it would be ∼0.04 and ∼0.09 mT for Gx and Gy readout gradients, if the B_0_ shift was assumed to be constantly applied for 0.1 ms. The second minor component was 10°–20° observed around the start of the readout gradients clearly seen in Fig. 5(c, f) as scattering points of the phase difference. This lasted for about 10 ms after the start of the data acquisition, corresponding to eddy currents with longer time constants.

### Data correction using the reference scan

As discussed above, the steady-state signal of the multiple gradient echo in the reference scan could be interpreted by a combination of B_0_ shifts and eddy current gradient fields with two time constants (∼0.3 and ∼2 ms). This result clearly supported the reference scan algorithm that the multiple gradient echoes were aligned by the resampling using the relative position of the echo peaks in the switching interval and the phases of the echoes were corrected using phases at the peaks of the gradient echoes. Therefore, the reference scan data can be used for other samples if the gradient coil set is placed exactly in the magnet bore in the same position as that of the reference scan measurement. The fact that the 3D EPI images of the celery were successfully corrected using the reference scan data obtained for the water phantom clearly shows the usefulness of the reference scan.

### Comparison of the size of the gradient coils

As shown in Table 2, the amplitudes were about two times larger than and the time constants of the “eddy current gradient field 2” were much longer (∼10 ms) than those of the standard size gradient-coil set. Therefore, the standard size gradient-coil set is essential for the development of EPI sequences using unshielded gradient coils in the conducting (Cu) room temperature bore.

### Magnetic field homogeneity and applications to real samples

A magnetic field induced in a spheroid made of homogeneous material is homogeneous when a homogeneous external magnetic field is applied parallel to the axis of the spheroid. However, because it is difficult to obtain a spheroidal material, samples with the long axis parallel to the magnetic field are used to obtain homogeneous magnetic field distribution around the middle of the samples. The water phantom used in this study and the stem of celery nearly met this condition. However, if the sample deviates from this condition, the magnetic field inhomogeneity would be large and the EPI image would be deformed. The image distortion can be corrected using magnetic field distribution measured in the 2D plane if the voxel size perpendicular to the 2D plane is made small using the 3D EPI approach.

## Conclusion

Several EPI sequences with 64 × 64, 128 × 128, 256 × 256, and 256 × 256 × 16 pixels were successfully developed in a 9.4 Tesla vertical standard bore superconducting magnet using a proper size unshielded gradient coil set optimized for live mice imaging. The positional and phase shifts of the multiple gradient echoes of the EPI signal caused by eddy currents were corrected using the reference scan technique. Therefore, we conclude that although our results may not be applied directly to a whole body MRI system, even at a high magnetic field such as 9.4 Tesla, EPI can be installed using a proper size unshielded gradient coil set, a higher order shim coil set, and careful data correction using the reference scan technique.

## Figures and Tables

**Table 1.** Parameters for eddy current gradient fields induced by current switching of the gradient coil with 39 and 32 mm outer and inner diameters. The amplitude means relative values of the induced gradient fields normalized by the applied gradient fields

	Eddy current gradient field 1		Eddy current gradient field 2	
	
	Amplitude (%)	Time constant (ms)	Amplitude (%)	Time constant (ms)
Gx	9.82	0.287	7.88	2.12
Gy	7.62	0.330	6.27	2.33
Gz	5.57	0.283	4.89	1.99

**Table 2.** Parameters for eddy current gradient fields induced by current switching of the gradient coil with 45 and 41 mm outer and inner diameters. The amplitude means relative values of the induced gradient fields normalized by the applied gradient fields

	Eddy current gradient field 1		Eddy current gradient field 2	
	
	Amplitude (%)	Time constant(ms)	Amplitude (%)	Time constant (ms)
Gx	20.1	2.12	14.6	12.7
Gz	20.4	1.48	19.7	9.70
